# Warts

**Published:** 1870-07

**Authors:** 


### WARTS.

If a bit of soft wood is chewed a little at one end, so as to make it brushy, and is then dipped into aqua fortis or nitric acid and rubbed lightly on the wart, so as to rub it in, it will disappear in a few days; the same is efficacious if applied to the warts on horses; some advise that the wart be shaved first, but we instinctively shudder at the idea, because warts, like corns, have been sometimes made to bleed excessively, causing lock-jaw and death.

